# Dietary Total Antioxidant Capacity, a Diet Quality Index Predicting Mortality Risk in US Adults: Evidence from the NIH-AARP Diet and Health Study

**DOI:** 10.3390/antiox12051086

**Published:** 2023-05-12

**Authors:** Kyungho Ha, Linda M. Liao, Rashmi Sinha, Ock K. Chun

**Affiliations:** 1 Department of Food Science and Nutrition, Jeju National University, Jeju 63243, Republic of Korea; kyungho.ha@jejunu.ac.kr; 2 Division of Cancer Epidemiology and Genetics, National Cancer Institute, Rockville, MD 20850, USA; linda.liao@nih.gov (L.M.L.); sinhar@mail.nih.gov (R.S.); 3 Department of Nutritional Sciences, University of Connecticut, Storrs, CT 06269, USA

**Keywords:** dietary total antioxidant capacity, diet quality, mortality, NIH-AARP Diet and Health Study

## Abstract

Dietary total antioxidant capacity (TAC) is an index representing the total antioxidant power of antioxidants consumed via the diet. This study aimed to investigate the association between dietary TAC and mortality risk in the US adults using data from the NIH-AARP Diet and Health Study. A total of 468,733 adults aged 50–71 years were included. Dietary intake was assessed using a food frequency questionnaire. Dietary TAC from diet was calculated from antioxidants including vitamin C, vitamin E, carotenoids, and flavonoids, and TAC from dietary supplements was calculated from supplemental vitamin C, vitamin E, and beta-carotene. During a median follow-up of 23.1 years, 241,472 deaths were recorded. Dietary TAC was inversely associated with all-cause (hazard ratio (HR) for quintile 5 vs. quintile 1: 0.97, 95% confidence interval (CI): 0.96–0.99, *p* for trend < 0.0001) and cancer mortality (HR for quintile 5 vs. quintile 1: 0.93, 95% CI: 0.90–0.95, *p* for trend < 0.0001). However, dietary supplement TAC was inversely associated with cancer mortality risk only. These findings indicate that consuming a habitual diet high in antioxidants may reduce the risk of all-cause and cancer mortality and TAC from foods might confer greater health benefits than TAC from dietary supplements.

## 1. Introduction

Dietary antioxidants help reduce oxidative stress and inflammation which can lead to several chronic diseases such as diabetes, cardiovascular diseases (CVDs) and cancer [1,2]. Antioxidants including carotenoids, vitamin C, vitamin E, and polyphenols such as flavonoids are concentrated in fruits, vegetables, coffee, tea, and wine [2] and previous epidemiologic studies reported that higher antioxidant intake from these foods is associated with lower mortality risk [3,4,5,6,7,8,9]. Notably, antioxidants have different antioxidant capacities and the combination of those found in foods that are part of our usual diet may have a cumulative or synergic effect [10,11].

Dietary total antioxidant capacity (TAC), which is a concept for assessing total antioxidant power of dietary antioxidants, has received attention due to its potential as a novel predictor of health outcomes. Although dietary TAC has been inversely associated with the risk of diabetes [12,13], CVDs [14,15,16,17], and cancer [18,19,20,21], a recent systematic review study concluded that the association between dietary TAC and mortality risk [22] was inconsistent, which may be due to discrepancies in methods of measuring TAC and consideration of supplemental antioxidant intakes.

Our research team has developed a simple theoretical algorithm that can estimate TAC from all food items in an individual’s diet [11]. This algorithm allows to evaluate dietary TAC just with dietary intake of individuals and has been validated using biomarkers [23,24] and through associations with clinical outcomes [19,25,26,27] and mortality [16,28] in US populations. This protocol was also applied in Korean populations [29] with associations between dietary TAC and diseases such as obesity and osteoporosis [30,31]. Recently, we evaluated the relative validity of dietary TAC compared to previous diet quality indexes, such as the Healthy Eating Index, alternate Mediterranean Diet, and the Dietary Approaches to Stop Hypertension by examining association between diet quality and all-cause mortality among US adults [28]. Therefore, dietary TAC estimated based on our protocols can be a useful diet quality index reflecting antioxidant-rich dietary patterns.

Nevertheless, due to a relatively small number of participants, short follow-up period, and exclusion of antioxidants from dietary supplements in previous studies [16,28], these associations need to be confirmed using data from a large-scale prospective cohort study. Thus, this analysis aimed to investigate the association between dietary TAC including from diet and dietary supplements and all-cause/cause-specific mortality using data from the NIH-AARP Diet and Health Study.

## 2. Materials and Methods

### 2.1. Study Population

The NIH-AARP Diet and Health Study is a large prospective cohort study designed to investigate the relationship between diet, lifestyle, and cancer risk. In 1995–1996, a baseline questionnaire assessing dietary intake and lifestyle habits was mailed to 3.5 million American adults aged 50–69 years who resided in one of six states (California, Florida, Pennsylvania, New Jersey, North Carolina, and Louisiana) or in two metropolitan areas (Atlanta, Georgia and Detroit, Michigan). Additional details regarding this study’s design have been described elsewhere [32]. Among 566,398 participants whose baseline data were available, we excluded those who were proxy-responders (*n* = 15,760), those who had a history of cancer (*n* = 51,346), those who reported implausible energy intakes (greater than two times the IQR below the 25th percentile or above the 75th percentile (*n* = 11,860), those with zero years of follow-up (*n* = 46), and those with a dietary TAC of greater than the 2IQR above the 75th percentile (*n* = 18,653). A total of 468,733 subjects were included in the final analysis.

### 2.2. Assessment of Dietary Total Antioxidant Capacity

Dietary TAC was calculated using self-reported diet and dietary supplement intake, respectively, according to the theoretical dietary TAC calculating algorithm [11]. This algorithm simply requires the participants’ antioxidant intakes and antioxidant capacity of each antioxidant compound. Baseline dietary intake was assessed using a 124-item semiquantitative food frequency questionnaire (FFQ) which asked about the intake of foods within the past year, and intakes of antioxidants including carotenoids (β-carotene, α-carotene, β-cryptoxanthin, lutein, zeaxanthin, and lycopene), vitamin C, vitamin E, and flavonoids (4 flavonols, 2 flavones, 3 flavanones, 10 flavan-3-ols, 4 isoflavones, and 6 anthocyanidins) were estimated. Flavonoid intakes were calculated by linking reported dietary intake to the USDA Expanded Flavonoid Database for the Assessment of Dietary Intakes, Release 1.1 [33]. Supplemental antioxidants included β-carotene, vitamin C, and vitamin E, and supplemental antioxidant intakes were assessed based on questions about vitamin supplement use. The antioxidant capacity as vitamin C equivalents (VCE) was previously measured using the 2,2′-azino-bis-3-ethylbenzthiazoline-6-sulphonic acid (ABTS) assay for each antioxidant [11]. For each participant, individual antioxidant capacities were calculated by multiplying the daily intake of individual antioxidants from diet and dietary supplements by their antioxidant capacities. Then, the individual antioxidant capacities were summed up to yield dietary TAC (diet + dietary supplements) [16].

### 2.3. Case Ascertainment

Participants were followed up from 1995–1996 until the date of death or the end of follow-up (31 December 2019), whichever came first. Vital status was ascertained using the National Death Index. All-cause mortality included mortality from cardiovascular disease (CVD) and cancer, as well as deaths from other circumstances. CVDs included diseases of the heart, hypertension without heart disease, cerebrovascular diseases, atherosclerosis, aortic aneurysm and dissection, and other diseases of arteries, arterioles, and capillaries. Cancer included cancers of oral cavity and pharynx, digestive system, respiratory system, soft tissue including heart, skin excluding basal and squamous, female genital system and breast, male genital system, urinary system, and endocrine system, lymphoma, leukemia, and miscellaneous cancer.

### 2.4. Assessment of Confounding Variables

Information on confounding variables including sociodemographic variables such as age, sex, race/ethnicity, education level, and marital status, and health-related variables such as body mass index (BMI), physical activity, alcoholic beverage intake, smoking, and history of disease was gathered from baseline questionnaires. Race/ethnicity was categorized into non-Hispanic White, non-Hispanic Black, Hispanic, and other. Education level was classified as less than 11 years, high school graduate, some college or other post-high school training, and college graduate. Marital status was categorized into married, widowed, divorced, or separated, and unmarried. Physical activity was defined as the frequency (never/rarely, 1–3 times/month, 1–2 times/week, 3–4 times/week, and ≥5 times/week) of exercise bouts that increase breathing or heart rate lasting ≥ 20 min in the past 12 months. Alcoholic beverage intake was categorized as none if patients reported never consuming alcohol, low if they reported a non-zero intake below the median among consumers, or high if they reported an intake above the median for consumers. Smoking status was defined as never, former, or current. History of heart disease, stroke, and diabetes was defined as yes or no.

### 2.5. Statistical Analysis

All statistical analyses were carried out using SAS version 9.4 (SAS Institute, Cary, NC, USA). Dietary TAC, including TAC from diet and supplements, was energy-adjusted using the residual method and was categorized into quintiles, except for TAC from supplements. For TAC from supplements, non-consumers were separated and then consumers were divided into tertiles. Differences in baseline characteristics of study participants according to quintiles of dietary TAC were evaluated using ANOVA for continuous variables or chi-square test for categorical variables. Cox proportional hazard regression models were used to estimate hazard ratios (HRs) and 95% confidence intervals (CIs) for mortality from all-cause, CVD, and cancer-related deaths according to quintiles of dietary TAC. Model 1 was adjusted for age (years, continuous) and sex, and Model 2 was additionally adjusted for race/ethnicity, body mass index (kg/m^2^, continuous), marital status, alcoholic beverage intake, smoking, physical activity, history of heart disease, stroke, and diabetes, and total energy intake (kcal/day, continuous) based on previous studies [34,35]. The Cox proportional hazard assumption was tested visually for covariates using Schoenfeld residual with no violations. Linear trends across quintiles were evaluated using the median TAC value of each quintile as a continuous variable. Restricted cubic splines were used to illustrate the association between dietary TAC and mortality risk, and HRs and 95% CIs were plotted for dietary TAC with the median value in the lowest quintile as the reference level. For dietary TAC from dietary supplements, zero value was used as the reference level because 32.6% of the subjects were non-consumers. Stratified analyses by age (<65 years/≥65 years), sex (male/female), body weight status (normal/obese), alcoholic beverage intake (none/low/high), and current smoking status (non-current/current) were conducted for TAC from diet only. All *p*-values were two-sided with α = 0.05 as the significance level.

## 3. Results

### 3.1. Baseline Characteristics

Baseline characteristics of study participants according to quintiles of energy-adjusted dietary TAC are presented in Table 1. Participants with higher dietary TAC tended to have lower BMI, higher education level, more frequent physical activity, and lower history of heart disease, stroke, and diabetes (*p* < 0.0001 for all). In addition, the proportions of subjects with high alcoholic beverage intake and those of current smokers were lowest in the highest quintile group of dietary TAC (*p* < 0.0001 for all).

### 3.2. Antioxidant Intakes and TAC

Table 2 shows antioxidant intakes and individual TAC levels from each antioxidant according to dietary TAC. TAC from diet was greater than TAC from supplements within the first and fourth quintile, whereas TAC from supplements was greater than TAC from diet in the highest quintile group. Antioxidants with high contribution to dietary TAC were flavonoids from diet and vitamin C from supplements.

### 3.3. Association between Dietary TAC and Mortality

During a median follow-up of 23.1 years, 241,472 deaths including 79,011 CVD and 65,733 cancer cases were recorded. Multivariable-adjusted HRs and 95% CIs for mortality according to quintiles of dietary TAC are shown in Table 3. In age- and sex-adjusted models (Model 1), dietary TAC was inversely associated with all-cause mortality (Q5 vs. Q1: HR = 0.83; 95% CI = 0.82–0.84; *p* for trend < 0.0001) as well as CVD (Q5 vs. Q1: HR = 0.87; 95% CI = 0.86–0.89; *p* for trend < 0.0001) and cancer mortality (Q5 vs. Q1: HR = 0.80; 95% CI = 0.78–0.82; *p* for trend < 0.0001). These significant inverse associations remained for cancer mortality in the fully adjusted models (Model 2) with HR of 0.93 (95% CI = 0.90–0.95; *p* for trend < 0.0001) for the highest quintile compared to the lowest quintile. When dividing TAC sources, TAC from diet was inversely associated with mortality risk, although there was no significant linear trend with CVD mortality. People in the highest tertile of TAC from supplements had a decreased cancer mortality risk (HR = 0.97; 95% CI = 0.95–0.99; *p* for trend = 0.0190), while they had an increased CVD mortality (HR = 1.03; 95% CI = 1.01–1.05; *p* for trend = 0.0002) compared to non-consumers in the fully adjusted models.

**Figure 1:**
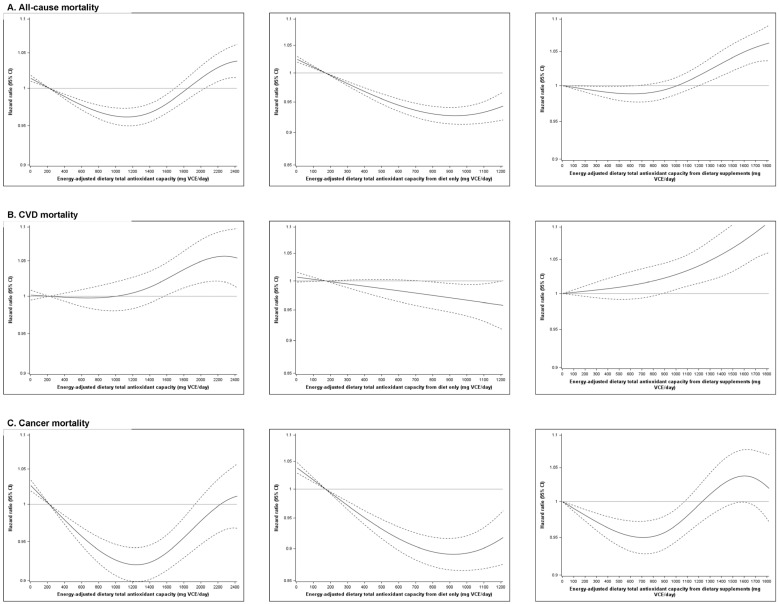
Hazard ratios from Cox proportional hazard regression models with restricted cubic spline curves describing the association between dietary TAC (from diet and dietary supplements (first column, reference level = 221.2 mg VCE/day), diet only (second column, reference level = 170.6 mg VCE/day), and dietary supplements only (third column, reference level = 0 mg VCE/day)) and all-cause (**A**), CVD (**B**), and cancer (**C**) mortality. Models included age, sex, race/ethnicity, body mass index, marital status, alcoholic beverage intake, smoking, physical activity, history of heart disease, stroke, and diabetes, and total energy intake.

For dietary TAC including diet and dietary supplements, there were U-shaped associations with all-cause and cancer mortality with lowest HRs at approximately 1200 mg VCE/day (Figure 1). The gradient of the inverse association between dietary TAC from diet only and all-cause and cancer mortality started to decrease at above approximately 800–900 mg VCE/day. There were positive associations between dietary TAC from dietary supplements and all-cause and CVD mortality at above 900–1200 mg VCE/day.

### 3.4. Stratified Analyses for Association between Dietary TAC from Diet Only and Mortality

For TAC from diet only, stratified analyses for mortality were conducted according to age, sex, body weight status, alcoholic beverage intake, and current smoking status (Table 4). Inverse association between TAC from diet and all-cause and cancer mortality were found in all subgroups except obese subjects for all-cause mortality. Regarding CVD mortality, significant inverse association with TAC from diet was detected in older, female, normal body weight, and non-current smoking subjects as well as subjects with none and high alcoholic beverage intake.

## 4. Discussion

This large-scale prospective cohort study found that dietary TAC from diet and dietary supplements was inversely associated with the risk of all-cause and cancer mortality. Additionally, people in the highest quintile of TAC from diet had reduced risk of all-cause, CVD, and cancer mortality compared to those in the lowest quintile. However, while people in the highest tertile of TAC from supplements had a decreased risk of cancer mortality, they had an increased risk of CVD mortality compared to non-consumers.

The inverse association between dietary TAC and all-cause mortality in the present study was in line with previous findings of studies on American [16,28], Spanish [3], Swedish [36], French [37], Japanese [17], and Chinese [14] populations, although no significant association was reported in Spanish elderly subjects at high cardiovascular risk [38]. A recent meta-analysis of five prospective studies also found significant inverse associations between dietary TAC and the risk of all-cause, CVD, and cancer mortality [39]. Considering that dietary TAC has been consistently associated with mortality regardless of methodological disparities in TAC assessments (e.g., ferric reducing antioxidant power (FRAP) and oxygen radical absorbance capacity (ORAC) assays), dietary TAC can be a valuable predictor of health outcomes such as mortality.

Notably, in this study, the associations of dietary TAC differed by the TAC sources, especially for CVD mortality. Dietary supplements were major sources of TAC in US adults [40], but effects of supplemental antioxidants on cardiovascular health are controversial. Several studies reported no significant effect of antioxidant supplements on the risk of CVD [41,42,43,44]. According to a recent meta-analysis of randomized controlled intervention trials, supplementation of vitamins C and E was not associated with CVD risk, while β-carotene supplementation was associated with increased all-cause mortality including CVD mortality [44]. Dietary and supplemental TAC showed consistent inverse relationships with cancer mortality in this study; however, in a prospective study based on the Prostate, Lung, Colorectal and Ovarian (PLCO) Cancer Screening Trial [18], a risk reduction of pancreatic cancer mortality was observed in TAC from diet, not TAC from supplements. In addition, antioxidant supplement use was not associated with cancer and non-cancer mortality based on the UK Biobank cohort study [45]. These findings indicate that the combination of antioxidants and synergistic effects of other compounds may be contained without the food matrix.

Study participants with higher dietary TAC consumed greater amounts of all types of antioxidants from diet and dietary supplements, which reflects a high diet quality. In our previous study, US adults with higher dietary TAC generally had higher adherence to diet quality indexes; they tended to consume greater amounts of whole fruits, dark green vegetables, whole grains, legumes, and nuts and seeds, as well as seafood [28]. Correlation between dietary TAC and diet quality has been confirmed in other studies as well [46,47,48]. Therefore, we cannot rule out the possibility that reduced risk of all-cause and cancer mortality according to dietary TAC might be resulted from overall healthy dietary pattern of people with high dietary TAC.

In a stratified analysis, TAC from diet was significantly inversely associated with cancer mortality in all subgroups. Significant inverse associations with CVD mortality were observed in older, female, and normal body weight, and in non-current smoking subjects as well as non-drinkers and those with high alcoholic beverage intakes. Although it is difficult to identify reasons for the differential associations with CVD mortality following stratification of participants on lifestyle factors in this study, one plausible explanation is that increased oxidative stress levels caused by obesity [49] and smoking [50] may attenuate the beneficial effect of dietary antioxidants on CVD.

Although there were statistically significant associations between dietary TAC and mortality risk, the effect size was relatively small compared to an increase in dietary TAC. For example, people in the highest quintile of dietary TAC had about seven times higher dietary TAC compared to those in the lowest quintile, but they had a 3% decreased risk of all-cause mortality. This might be partly due to the wide range of dietary TAC with right-skewed distributions, and therefore the associations should be interpreted with changes in dietary TAC. Moreover, restricted cubic spline curves indicated that there were U-shaped associations between dietary TAC and all-cause and cancer mortality and the gradient of the inverse association between TAC from diet only and all-cause and cancer mortality decreased from above a specific point. Further studies are required to investigate the optimal levels of dietary antioxidants and TAC with considerations of serum antioxidants, bioavailability of antioxidants, and interaction with other nutrients.

To the best of our knowledge, this is the first study investigating the association between theoretical dietary TAC from diet and dietary supplements and mortality from all causes, CVD, and cancer using data from a large-scale prospective study with a long follow-up. However, this study has some limitations. First, dietary intake measured at single point (baseline) may not reflect the cumulative average intake. Although dietary TAC might have changed over time due to changes in eating behavior, food supply, and/or perceptions of what is considered healthy, according to studies with long-term prospective cohorts, the dietary intake data obtained from the FFQ at baseline reflect longer-term dietary quality and are linked to diet-related health risk [51,52,53]. In addition, using only the earliest dietary measure may be appropriate to examine hypothetical associations between dietary exposures and diseases with a long latency [54]. Second, dietary TAC estimated in this study does not reflect an individual’s bioavailability of antioxidants; however, we previously validated its correlation with a body’s antioxidant status in populations with various physiological conditions [25,55]. Third, dietary TAC may be underestimated because proanthocyanidines and other polyphenols were not calculated in this study. Fourth, because many of the participants were non-Hispanic white, it may be difficult to generalize the findings to other populations with different race/ethnicity. Lastly, there might be unmeasured residual confounding.

## 5. Conclusions

This study found that dietary TAC from diet and dietary supplements was associated with mortality risk using data from a large longitudinal study. Although both TAC from diet and supplements may decrease all-cause and cancer-related deaths, TAC from foods might confer greater health benefits than TAC from dietary supplements. Further studies are required to establish recommendations of appropriate levels of dietary antioxidants such as flavonoids and TAC.

## Figures and Tables

**Table 1:** Baseline characteristics of study participants according to energy-adjusted dietary total antioxidant capacity.

Characteristics	Energy-Adjusted Dietary Total Antioxidant Capacity (Diet + Dietary Supplements)					
	Quintile 1 (*n* = 93,746)	Quintile 2 (*n* = 93,747)	Quintile 3 (*n* = 93,747)	Quintile 4 (*n* = 93,747)	Quintile 5 (*n* = 93,746)	*p* Value
Age (years)	61.3 ± 5.4 ^1^	61.6 ± 5.4	61.6 ± 5.4	61.7 ± 5.4	61.5 ± 5.3	<0.0001
BMI (kg/m^2^)	27.5 ± 5.1	27.3 ± 5.0	27.2 ± 5.1	26.9 ± 5.0	26.5 ± 5.1	<0.0001
Energy intake (kcal/day)	1688.7 ± 718.1	1782.2 ± 697.2	1850.7 ± 711.6	1833.2 ± 689.5	1639.0 ± 640.4	<0.0001
Sex						<0.0001
Male	62,626 (66.8)	58,054 (61.9)	55,475 (59.2)	53,540 (57.1)	48,668 (51.9)	
Female	31,120 (33.2)	35,693 (38.1)	38,272 (40.8)	40,207 (42.9)	45,078 (48.1)	
Race/ethnicity						<0.0001
Non-Hispanic White	85,786 (93.0)	86,061 (93.0)	84,796 (91.7)	84,936 (91.9)	86,599 (93.5)	
Non-Hispanic Black	3485 (3.8)	3672 (4.0)	4188 (4.5)	3925 (4.3)	2595 (2.8)	
Hispanic	1791 (1.9)	1643 (1.8)	1886 (2.0)	1828 (2.0)	1735 (1.9)	
Other	1196 (1.3)	1184 (1.3)	1624 (1.8)	1722 (1.9)	1668 (1.8)	
Education						<0.0001
Less than 11 years	7431 (8.2)	5792 (6.4)	5426 (6.0)	5284 (5.8)	3673 (4.0)	
High school graduate	21,687 (23.9)	19,172 (21.0)	18,275 (20.1)	17,631 (19.4)	14,838 (16.3)	
Some college or other post-HS training	31,917 (35.1)	30,674 (33.6)	30,723 (33.8)	30,573 (33.7)	30,986 (34.0)	
College graduate	29,853 (32.9)	35,588 (39.0)	36,561 (40.2)	37,310 (41.1)	41,553 (45.6)	
Marital status						<0.0001
Married	67,319 (72.6)	66,503 (71.5)	64,443 (69.3)	63,113 (67.9)	60,051 (64.5)	
Widowed, divorced, or separated	21,492 (23.2)	22,303 (24.0)	24,036 (25.9)	25,174 (27.1)	28,129 (30.2)	
Unmarried	3931 (4.2)	4230 (4.6)	4496 (4.8)	4676 (5.0)	4932 (5.3)	
Physical activity ^2^						<0.0001
Never/rarely	23,095 (25.0)	17,286 (18.6)	16,076 (17.4)	14,875 (16.0)	13,109 (14.1)	
1–3 times/mo	15,224 (16.5)	13,361 (14.4)	12,681 (13.7)	11,664 (12.6)	10,975 (11.8)	
1–2 times/wk	20,248 (21.9)	21,030 (22.7)	20,457 (22.1)	20,217 (21.8)	19,172 (20.6)	
3–4 times/wk	20,475 (22.2)	24,709 (26.7)	25,538 (27.6)	26,681 (28.8)	28,176 (30.3)	
≥5 times/wk	13,336 (14.4)	16,338 (17.6)	17,906 (19.3)	19,273 (20.8)	21,535 (23.2)	
Alcoholic beverage intake (g/day) ^3^						<0.0001
None	45,378 (48.4)	43,092 (46.0)	43,620 (46.5)	43,563 (46.5)	44,492 (47.5)	
Low	21,907 (23.4)	24,256 (25.9)	25,013 (26.7)	25,300 (27.0)	25,245 (26.9)	
High	26,461 (28.2)	26,399 (28.2)	25,114 (26.8)	24,884 (26.5)	24,009 (25.6)	
Smoking						<0.0001
Never	27,304 (30.3)	32,620 (36.2)	34,740 (38.6)	35,128 (38.9)	34,667 (38.4)	
Former	46,171 (51.3)	46,963 (52.0)	45,523 (50.5)	45,511 (50.5)	46,877 (51.9)	
Current	16,532 (18.4)	10,657 (11.8)	9835 (10.9)	9563 (10.6)	8832 (9.8)	
History of heart disease						<0.0001
No	80,430 (85.8)	80,351 (85.7)	80,781 (86.2)	80,885 (86.3)	81,261 (86.7)	
Yes	13,316 (14.2)	13,396 (14.3)	12,966 (13.8)	12,862 (13.7)	12,485 (13.3)	
History of stroke						<0.0001
No	91,455 (97.6)	91,767 (97.9)	91,792 (97.9)	91,860 (98.0)	92,094 (98.2)	
Yes	2291 (2.4)	1980 (2.1)	1955 (2.1)	1887 (2.0)	1652 (1.8)	
History of diabetes						<0.0001
No	83,932 (89.5)	84,622 (90.3)	85,179 (90.9)	85,707 (91.4)	86,758 (92.6)	
Yes	9814 (10.5)	9125 (9.7)	8568 (9.1)	8040 (8.6)	6988 (7.5)	

^1^ All values are presented as mean ± SD or *n* (%). ^2^ Physical activities lasted for ≥20 min in the past 12 months that caused increases in breathing or heart rate or worked up a sweat. ^3^ Alcoholic beverage intake was divided equally in half (low or high) among alcoholic beverage drinkers.

**Table 2:** Antioxidant intakes according to energy-adjusted dietary total antioxidant capacity.

Antioxidant	Energy-Adjusted Dietary Total Antioxidant Capacity (Diet + Dietary Supplements)				
	Quintile 1 (*n* = 93,746)	Quintile 2 (*n* = 93,747)	Quintile 3 (*n* = 93,747)	Quintile 4 (*n* = 93,747)	Quintile 5 (*n* = 93,746)
From diet					
Alpha-carotene (μg)	589.7 ± 731.7	858.6 ± 1018.5	1012.8 ± 1209.6	1063.1 ± 1305.6	1117.9 ± 1418.1
Beta-carotene (μg)	2630.2 ± 2161	3786 ± 3043.7	4503.7 ± 3757	4705.9 ± 4178.3	4822.1 ± 4353.8
Beta-cryptoxanthin (μg)	90.1 ± 59.8	165.8 ± 93.4	221.1 ± 140.3	237 ± 189.5	224.1 ± 177.3
Lutein + zeaxanthin (μg)	1920.3 ± 1559.4	2728.6 ± 2278.8	3251.6 ± 2876	3371.8 ± 3221.8	3379.9 ± 3247.2
Lycopene (μg)	5568.7 ± 4430.5	6895.8 ± 5641.2	7705.9 ± 6984	7802.4 ± 7749.8	7426.8 ± 7454.4
Vitamin E (mg)	6.3 ± 3.1	7.1 ± 3.4	7.5 ± 3.5	7.5 ± 3.5	6.9 ± 3.3
Vitamin C (mg)	81.5 ± 38.5	136.2 ± 57.4	174.9 ± 87.2	184.6 ± 116.7	175.4 ± 109.4
Flavonoids (mg)	54.6 ± 25.3	103.4 ± 36.6	154.3 ± 62.5	179.7 ± 109.5	176.3 ± 117.5
Flavonols	11.9 ± 6.8	17.1 ± 8.2	22.4 ± 10.4	24.9 ± 14.3	24.5 ± 14.9
Flavones	0.6 ± 0.4	0.9 ± 0.6	1.1 ± 0.9	1.2 ± 1.0	1.2 ± 1.0
Flavanones	15.4 ± 14.4	34.8 ± 24.4	49.5 ± 37	53.8 ± 50.9	50.3 ± 47.6
Flavan-3-ols	19.5 ± 14.3	38.3 ± 29.7	65.6 ± 51.7	83.5 ± 86.7	83.7 ± 95.8
Anthocyanidins	6.8 ± 6.7	11.8 ± 10.9	15.3 ± 15.2	16.0 ± 17.2	16.1 ± 16.4
Isoflavones	0.4 ± 0.3	0.4 ± 0.3	0.4 ± 0.3	0.4 ± 0.3	0.3 ± 0.3
From dietary supplements					
Beta-carotene (μg)	166.1 ± 379.9	335.5 ± 527.3	494.6 ± 695.0	886.0 ± 1125.7	1525.8 ± 1626.4
Vitamin E (a-TE)	11.7 ± 40.7	29.4 ± 67.2	48.5 ± 83.6	93.7 ± 99.5	168.7 ± 119.9
Vitamin C (mg)	15.3 ± 29.6	40.1 ± 55.2	99.8 ± 129.7	351.6 ± 250	982.1 ± 420.8
Total antioxidant capacity (mg VCE)	205.0 ± 73.6	375.4 ± 79.7	574.6 ± 123.3	899.8 ± 182.1	1537.9 ± 379.9
From diet	186.4 ± 73.2	327.1 ± 97.4	461.4 ± 162.5	522.2 ± 273.6	509.0 ± 283.9
From carotenoids	4.5 ± 3.0	5.8 ± 3.9	6.6 ± 4.8	6.7 ± 5.4	6.5 ± 5.2
From vitamin E	1.7 ± 0.9	2.0 ± 0.9	2.1 ± 1.0	2.1 ± 1.0	1.9 ± 0.9
From vitamin C	81.5 ± 38.5	136.2 ± 57.4	174.9 ± 87.2	184.6 ± 116.7	175.4 ± 109.4
From flavonoids	98.8 ± 46.3	183.2 ± 73.6	277.9 ± 126.9	328.8 ± 222.2	325.2 ± 242.8
From dietary supplements	18.6 ± 33.8	48.3 ± 61.6	113.2 ± 136.8	377.6 ± 261.6	1028.8 ± 432.1
From beta-carotene	0.04 ± 0.10	0.08 ± 0.13	0.12 ± 0.18	0.22 ± 0.28	0.38 ± 0.41
From vitamin E	3.2 ± 11.2	8.1 ± 18.5	13.3 ± 23.0	25.8 ± 27.4	46.4 ± 33.0
From vitamin C	15.3 ± 29.6	40.1 ± 55.2	99.8 ± 129.7	351.6 ± 250	982.1 ± 420.8

**Table 3:** Hazard ratios and 95% confidence intervals of mortality according to energy-adjusted dietary total antioxidant capacity.

	Energy-Adjusted Dietary Total Antioxidant Capacity (Diet + Dietary Supplements)					
	Quintile 1 (*n* = 93,746)	Quintile 2 (*n* = 93,747)	Quintile 3 (*n* = 93,747)	Quintile 4 (*n* = 93,747)	Quintile 5 (*n* = 93,746)	*p* for Trend
Median (range)	221.2 (8.9–303)	378.9 (303–462.4)	564.9 (462.4–707.8)	893.7 (707.8–1132.4)	1543.3 (1132.4–5926.6)	
All-cause mortality						
Model 1 ^1^	1.00	0.88 (0.87–0.89)	0.85 (0.84–0.86)	0.85 (0.84–0.86)	0.83 (0.82–0.84)	<0.0001
Model 2 ^2^	1.00	0.96 (0.94–0.97)	0.95 (0.94–0.96)	0.96 (0.95–0.97)	0.97 (0.96–0.99)	0.1688
CVD mortality						
Model 1	1.00	0.90 (0.88–0.92)	0.88 (0.86–0.90)	0.88 (0.86–0.90)	0.87 (0.86–0.89)	<0.0001
Model 2	1.00	0.97 (0.95–0.996)	0.98 (0.95–1.00)	0.99 (0.97–1.01)	1.02 (0.99–1.04)	0.0043
Cancer mortality						
Model 1	1.00	0.85 (0.83–0.87)	0.83 (0.81–0.85)	0.82 (0.80–0.84)	0.80 (0.78–0.82)	<0.0001
Model 2	1.00	0.93 (0.91–0.95)	0.92 (0.90–0.95)	0.92 (0.89–0.94)	0.93 (0.90–0.95)	<0.0001
	Energy-adjusted total antioxidant capacity from diet only					
	Quintile 1 (*n* = 93,746)	Quintile 2 (*n* = 93,747)	Quintile 3 (*n* = 93,747)	Quintile 4 (*n* = 93,747)	Quintile 5 (*n* = 93,746)	*p* for trend
Median (range)	170.6 (8–225.4)	272 (225.4–316.3)	362.5 (316.3–414.4)	477.6 (414.4–562.8)	709.4 (562.8–3908.3)	
All-cause mortality						
Model 1	1.00	0.85 (0.84–0.86)	0.81 (0.80–0.82)	0.80 (0.79–0.81)	0.80 (0.79–0.81)	<0.0001
Model 2	1.00	0.93 (0.92–0.94)	0.92 (0.91–0.93)	0.92 (0.90–0.93)	0.93 (0.92–0.94)	<0.0001
CVD mortality						
Model 1	1.00	0.87 (0.85–0.89)	0.84 (0.82–0.86)	0.84 (0.83–0.86)	0.85 (0.84–0.87)	<0.0001
Model 2	1.00	0.94 (0.92–0.97)	0.93 (0.91–0.95)	0.95 (0.93–0.98)	0.97 (0.94–0.99)	0.2122
Cancer mortality						
Model 1	1.00	0.82 (0.80–0.84)	0.78 (0.77–0.80)	0.76 (0.74–0.78)	0.76 (0.75–0.78)	<0.0001
Model 2	1.00	0.91 (0.89–0.94)	0.90 (0.88–0.92)	0.88 (0.86–0.91)	0.90 (0.88–0.92)	<0.0001
	Energy-adjusted total antioxidant capacity from dietary supplements only					
	Non-consumer (*n* = 153,024)	Tertile 1 (*n* = 105,236)	Tertile 2 (*n* = 105,237)	Tertile 3 (*n* = 105,236)	*p* for trend	
Median (range)	0 (0–0)	58.2 (0–103.3)	276.5 (103.3–555)	938 (555–3345.8)		
All-cause mortality						
Model 1	1.00	0.95 (0.94–0.96)	0.90 (0.89–0.91)	0.92 (0.91–0.93)	<0.0001	
Model 2	1.00	1.00 (0.99–1.01)	0.99 (0.98–0.998)	1.01 (0.99–1.02)	0.2513	
CVD mortality						
Model 1	1.00	0.92 (0.90–0.94)	0.91 (0.90–0.93)	0.93 (0.91–0.94)	<0.0001	
Model 2	1.00	0.98 (0.96–0.997)	1.01 (0.99–1.03)	1.03 (1.01–1.05)	0.0002	
Cancer mortality						
Model 1	1.00	0.95 (0.93–0.97)	0.89 (0.87–0.90)	0.91 (0.89–0.93)	<0.0001	
Model 2	1.00	0.99 (0.96–1.01)	0.94 (0.92–0.96)	0.97 (0.95–0.99)	0.0190	

^1^ Model 1 included age and sex. ^2^ Model 2 additionally included race/ethnicity, body mass index, marital status, alcoholic beverage intake, smoking, physical activity, history of heart disease, stroke, and diabetes, and total energy intake.

**Table 4:** Stratified analysis for mortality associated with energy-adjusted total antioxidant capacity from diet only.

Subgroup	N	Hazard Ratio (95% Confidence Interval) ^1,2^		
		All-Cause Mortality	CVD Mortality	Cancer Mortality
Age				
<65 years	300,678	0.95 (0.93–0.96) ^1^	1.00 (0.97–1.04)	0.91 (0.88–0.94)
≥65 years	168,055	0.91 (0.89–0.93)	0.94 (0.91–0.97)	0.89 (0.85–0.92)
P for interaction		<0.0001	0.0739	0.0034
Sex				
Male	278,363	0.93 (0.92–0.95)	0.98 (0.95–1.00)	0.88 (0.86–0.91)
Female	190,370	0.93 (0.91–0.95)	0.95 (0.91–0.99)	0.93 (0.89–0.97)
P for interaction		0.8142	0.4384	0.4666
Body weight status				
Normal	357,252	0.91 (0.90–0.92)	0.94 (0.92–0.97)	0.89 (0.87–0.92)
Obese	100,302	1.00 (0.97–1.03)	1.05 (1.00–1.09)	0.94 (0.89–0.99)
P for interaction		<0.0001	<0.0001	0.1175
Alcoholic beverage intake				
None	220,145	0.93 (0.91–0.95)	0.96 (0.92–0.99)	0.91 (0.88–0.95)
Low	121,721	0.96 (0.93–0.98)	1.01 (0.96–1.06)	0.92 (0.88–0.97)
High	126,867	0.91 (0.89–0.93)	0.95 (0.90–0.99)	0.87 (0.83–0.91)
P for interaction		0.2184	0.0763	0.2140
Current smoking status				
Never/former smokers	395,504	0.92 (0.91–0.94)	0.95 (0.93–0.98)	0.89 (0.86–0.91)
Current smokers	55,419	0.90 (0.87–0.94)	0.99 (0.93–1.06)	0.88 (0.83–0.93)
P for interaction		0.4675	0.2773	0.0732

^1^ Adjusted for age, sex (except for sex-stratified analysis), race/ethnicity, body mass index (except for body weight status-stratified analysis), marital status, alcoholic beverage intake (except for alcoholic beverage intake-stratified analysis), smoking (except for current smoking status-stratified analysis), physical activity, history of heart disease, stroke, and diabetes, and total energy intake. ^2^ Hazard ratios of quintile 5 compared to quintile 1 were presented.

## Data Availability

Data were obtained from the National Cancer Institute and are available for established researchers by submitting a project proposal to https://www.nihaarpstars.com (accessed on 29 June 2020).
